# Control of Gene Expression by Exosome-Derived Non-Coding RNAs in Cancer Angiogenesis and Lymphangiogenesis

**DOI:** 10.3390/biom11020249

**Published:** 2021-02-09

**Authors:** Valeria Arcucci, Steven A. Stacker, Marc G. Achen

**Affiliations:** 1 Tumour Angiogenesis and Microenvironment Program, Peter MacCallum Cancer Centre, 305 Grattan St., Melbourne VIC 3000, Australia; valeria.arcucci@petermac.org (V.A.); steven.stacker@petermac.org (S.A.S.); 2 Sir Peter MacCallum Department of Oncology, University of Melbourne, Parkville VIC 3010, Australia; 3 Department of Surgery, Royal Melbourne Hospital, The University of Melbourne, Parkville VIC 3050, Australia; 4 O’Brien Institute Department, St Vincent’s Institute of Medical Research, 9 Princes Street, Fitzroy VIC 3065, Australia; 5 Department of Medicine, St Vincent’s Hospital, University of Melbourne, Fitzroy VIC 3065, Australia

**Keywords:** non-coding RNA, microRNA, exosomes, cancer, blood vessels, lymphatic vessels

## Abstract

Tumour angiogenesis and lymphangiogenesis are hallmarks of cancer and have been associated with tumour progression, tumour metastasis and poor patient prognosis. Many factors regulate angiogenesis and lymphangiogenesis in cancer including non-coding RNAs which are a category of RNAs that do not encode proteins and have important regulatory functions at transcriptional and post-transcriptional levels. Non-coding RNAs can be encapsulated in extracellular vesicles called exosomes which are secreted by tumour cells or other cells in the tumour microenvironment and can then be taken up by the endothelial cells of blood vessels and lymphatic vessels. The “delivery” of these non-coding RNAs to endothelial cells in tumours can facilitate tumour angiogenesis and lymphangiogenesis. Here we review recent findings about exosomal non-coding RNAs, specifically microRNAs and long non-coding RNAs, which regulate tumour angiogenesis and lymphangiogenesis in cancer. We then focus on the potential use of these molecules as cancer biomarkers and opportunities for exploiting ncRNAs for the treatment of cancer.

## 1. Introduction

The growth of new tumour blood vessels and lymphatic vessels is a key element of cancer progression having a close association with metastatic spread, poor patient prognosis and survival [1,2]. Tumour angiogenesis is a well-established target for anti-cancer therapeutics through targeting of growth factors, their cell surface receptors and associated signalling pathways [3], however, tumour lymphangiogenesis is yet to be clinically validated as a therapeutic target. A deeper knowledge of the array of molecular mechanisms controlling tumour angiogenesis and lymphangiogenesis will be central to development of novel therapeutics for targeting these processes in cancer. 

**Figure 1:**
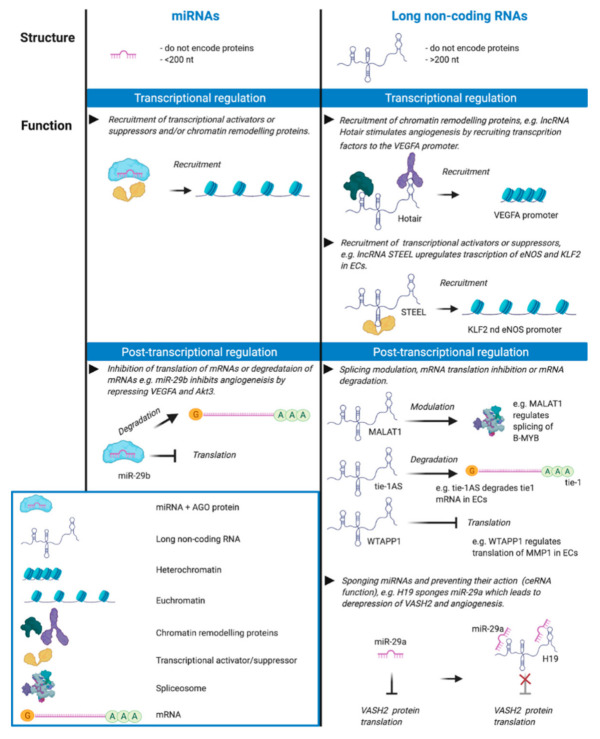
Non-coding RNA is classified as either long or small based on a 200-nucleotide cut-off. Small RNA (e.g., miRNA) has less defined secondary structure whereas long non-coding RNA (lncRNA) has more defined secondary structure required for its function in transcriptional regulation by binding chromatin remodelling proteins and recruitment of transcription factors, activators and suppressors. For example, lncRNA STEEL, an endothelial cell-specific lncRNA, upregulates transcription of the genes for endothelial nitric oxide synthase (eNOS) and the transcription factor Kruppel-like factor 2 (KLF2) by recruiting PARP1 to their promoter regions [9]. lncRNA Hotair stimulates angiogenesis by recruiting chromatin remodelling proteins which activate transcription of the gene for vascular endothelial growth factor A (VEGFA) [10]. Nuclear miRNAs play a role in transcription by recruiting transcriptional activator and repressor chromatin remodelling proteins [6], and miRNAs are involved in the post-transcriptional regulation of mRNAs by modulating their translation or degradation, e.g., miR-29-b inhibits angiogenesis by negatively regulating the expression of VEGFA and Akt3 [11,12]. lncRNAs can act post-transcriptionally to regulate mRNA by modulating mRNA splicing, translation and mRNA degradation, e.g., lncRNA MALAT1 plays a role in angiogenesis through several mechanisms including the regulation of alternative splicing of the oncogenic transcription factor B-MYB in endothelial cells [13], WTAPP1 lncRNA promotes migration by increasing the expression of the matrix metalloproteinase MMP1 [14] and Tie-1AS lncRNA selectively binds and degrades tie-1 mRNA leading to specific defects in cell junctions and tube formation [15]. Further, lncRNAs can act as sponges to bind and sequester miRNAs, e.g., lncRNA H19 sponges miR-29a resulting in derepression of Vasohibin 2 (VASH2) and angiogenesis [16]. This figure was created with BioRender.com.

Previous work has shown that a range of growth factors, cell surface receptors and a multitude of signalling molecules drives the remodelling of the blood and lymphatic vasculatures in cancer [2,4]. Recent research has identified important roles for non-coding RNAs (ncRNAs) in regulating key aspects of cancer biology, including tumour angiogenesis and lymphangiogenesis. ncRNAs are a class of RNA molecules which do not encode proteins; they are classified as “small” (<200 nucleotides) or “long” (>200 nucleotides) based uniquely on their length [5]. The most studied type of small ncRNAs are microRNAs (miRNAs) which, together with long ncRNAs, are the primary focus of this review. miRNAs are small RNA molecules which mediate post-transcriptional regulation by targeting mRNAs leading to decreased gene expression (i.e., gene silencing) via mRNA degradation and/or translational repression. Nuclear miRNAs have been shown to play a role in the regulation of transcription by recruiting transcriptional activator and repressor chromatin remodelling proteins (Figure 1) [6,7]. Importantly, different miRNAs can work cooperatively in miRNA clusters to convergently target expression of the same or multiple genes in related molecular pathways [8].

Long ncRNAs (lncRNAs) exhibit a range of different regulatory functions in distinct cellular compartments [5] (Figure 1). They can bind RNA, DNA and proteins, and regulate gene expression at several different levels. They act as transcriptional regulators by recruiting transcriptional activators or suppressors [17,18] or by recruiting chromatin remodelling proteins, thereby favouring epigenetic control of gene expression [19]. lncRNAs can also regulate gene expression at a post-transcriptional level by recruiting proteins to degrade mRNAs [20], modulating splicing of mRNAs [21] or acting as decoys for proteins involved in mRNA degradation [22]. lncRNAs can also regulate miRNA function: some lncRNAs act as competing endogenous RNAs which sequester multiple miRNAs thereby inhibiting their action leading to de-repression of certain mRNA targets [23]. 

An important functional feature of miRNAs and lncRNAs is that they can exert effects on the regulation of complex biological responses: miRNAs by targeting mRNAs encoding multiple proteins involved in the same or a related molecular pathway, lncRNAs by controlling the remodelling of chromatin to modulate gene expression, or by targeting miRNAs. Moreover, a miRNA can act in concert with other miRNAs, lncRNAs or transcription factors to mediate gene silencing in a precise manner.

It has recently been shown that ncRNAs can be encapsulated in extracellular vesicles called exosomes and thus take on a pivotal role in cell-to-cell communication which occurs in the tumour microenvironment and ultimately contributes to tumour progression, remodelling of blood vessels and lymphatics, and tumour spread [24,25]. In this review, we summarise recent discoveries about ncRNAs, both miRNAs and lncRNAs, which are secreted by cancer cells in exosomes and which then facilitate tumour angiogenesis and lymphangiogenesis. Further, we discuss the use of these tumour-derived exosomal ncRNAs as tumour biomarkers, and the potential of exploiting ncRNAs for treating cancer. 

## 2. Tumour Angiogenesis and Lymphangiogenesis

### 2.1. Tumour Angiogenesis

Tumour cells require oxygen and nutrients to proliferate, and solid tumours therefore require blood vessels to facilitate their growth and spread [26]. Tumour cells and other cells belonging to the tumour microenvironment can secrete angiogenic signalling molecules that trigger the so called “angiogenic switch” which is the transition from a non-vascular to a vascularised tumour phenotype [1]. The formation of blood vessels in tumours occurs through several different processes: new capillaries can sprout from pre-existing ones (sprouting angiogenesis) [27], a new vessel can form to connect two pre-existing vessels or new blood vessels can form *de novo* from endothelial progenitor cells or cancer stem cells in a process called vasculogenesis [28]. Tumour blood vessels appear chaotic and disorganised, having endothelial cell junctions which are often disrupted, leading to increased vascular permeability and interstitial fluid pressure. This can reduce the efficacy of cancer therapy since compression of tumour vessels inhibits drug delivery [29]. Several angiogenic factors, produced by tumour cells and/or other cells in the tumour microenvironment, promote tumour angiogenesis and will be described in the last part of this section.

### 2.2. Tumour Lymphangiogenesis

Lymphatic vessels can undergo multiple changes in cancer which facilitate the spread of tumour cells to organs distant from the primary tumour, such as lymphangiogenesis and other forms of lymphatic remodelling [2]. Lymphangiogenesis, the formation of new lymphatic vessels from the pre-existing lymphatic vascular network, involves proliferation, sprouting and migration of lymphatic endothelial cells (LECs) to form new tumoural lymphatic vessels. Tumour lymphangiogenesis correlates with the spread of tumour cells to lymph nodes in mice [30,31,32]. An alternative form of lymphatic remodelling, lymphatic enlargement, occurs in the large collecting lymphatic vessels and involves morphological changes that lead to the dilation of these vessels which is associated with increased lymph node metastasis [33,34,35]. 

The remodelling of the lymphatic vasculature in cancer is driven by a diverse range of growth factors that are produced by tumour cells and cells in the tumour microenvironment. These growth factors bind cognate receptors on LECs and activate several molecular pathways that ultimately drive lymphangiogenesis; they are described below. 

### 2.3. Molecular Regulation of Angiogenesis and Lymphangiogenesis in Cancer

#### 2.3.1. VEGF Signalling Pathways in Angiogenesis and Lymphangiogenesis

Some members of the vascular endothelial growth factor (VEGF) family of secreted proteins are inducers of angiogenesis and lymphangiogenesis in cancer. The human VEGF family is composed of VEGFA, VEGFB, VEGFC, VEGFD and placenta growth factor (PIGF). These growth factors bind and activate cognate receptors VEGFR1, VEGFR2 and VEGFR3 with different affinities [36,37,38,39,40,41]. VEGFR2 and VEGFR3 are mainly expressed on endothelial cells [42] while VEGFR1, which is also expressed on endothelial cells, has in addition been shown to be expressed on macrophages and to regulate their migration [43].

The VEGFC-VEGFR3 and VEGFD-VEGFR3 signalling pathways have been the most studied in lymphangiogenesis and lymphatic vessel remodelling in cancer and are considered major drivers of these processes [2]. In a tumour context, the lymphangiogenic growth factors VEGFC and VEGFD are produced by a variety of cell types including tumour cells, stromal cells, tumour-infiltrating macrophages and activated platelets [44,45]. Binding of VEGFC and VEGFD to VEGFR3 on LECs causes receptor homodimerization and autophosphorylation, leading to the activation of downstream lymphangiogenic signalling involving the RAS-MAPK and PI3K-AKT pathways [46].

VEGFA is secreted by tumour cells and stroma and is the most studied inducer of tumour angiogenesis; its presence is correlated with tumour size, blood vessel density and metastasis [47]. VEGFA is upregulated in hypoxic conditions, which are a hallmark of most solid tumours [48], and is thought to act mainly by activating VEGFR2 on the surface of blood vascular endothelial cells (BECs) [47]. 

#### 2.3.2. Other Signalling Pathways

Besides the VEGFs, other growth factors and signalling pathways have been reported to stimulate lymphangiogenesis and angiogenesis. Angiopoietins 1 and 2 (ANGPT1 and ANGPT2) and their receptors (TIE1 and TIE2) on endothelial cells are required for both these processes [49]. Fibroblast growth factor 2 (FGF2), a member of the FGF family [50], and platelet-derived growth factor B [51] have been proposed to exhibit lymphangiogenic activity. Several other factors may also play a role in tumour lymphangiogenesis such as Hepatocyte growth factor [52], Insulin-like growth factors [53], Epidermal growth factor [54] and Transforming growth factor β [55]. In terms of angiogenic signalling, members of the Eph/ephrin transmembrane protein families have been shown to promote tumour angiogenesis, as well as tumour progression, and to impair anti-angiogenic therapies [56].

Lymphangiogenesis and angiogenesis in tumours involve complex regulation by stimulatory molecules which modulate interacting signalling pathways to induce endothelial cell proliferation, sprouting, migration and eventually formation of new vessels from the pre-existing network. It is clear from the literature that the proteins which are best validated as lymphangiogenic factors in vivo, acting directly on LEC, are VEGFC and VEGFD. Likewise, VEGFA is the best validated angiogenic factor in cancer. These conclusions are based on many mouse genetic models, tumour xenograft models and clinicopathological studies [2,4,57]. 

## 3. Role of Exosome-Derived Non-Coding RNAs in Tumour Lymphangiogenesis and Angiogenesis

**Figure 2:**
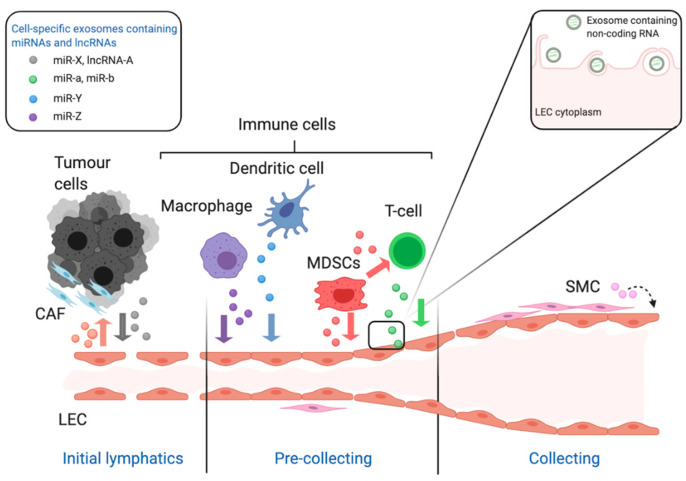
The schematic diagram illustrates the role of non-coding RNA in the growth and remodelling of lymphatic vessels in the context of cancer. The lymphatic system is composed of a hierarchy of vessels starting with blind-ended initial lymphatics which allow the entry of cells and fluid via their incomplete junctions. Lymph fluid then flows through more structured pre-collecting vessels to collecting vessels which acquire a complete lining of smooth muscle cells. All of these lymphatic vessels are lined with specialised lymphatic endothelial cells (LEC). In the context of cancer miRNA and long non-coding RNA are released from tumour cells and cells of the tumour microenvironment via exosomes. This includes but is not limited to tumour cells, macrophages, dendritic cells, T-lymphocytes and smooth muscle cells. These cells release exosomes containing specific miRNAs and lncRNAs, which is indicated in the legend and by the coloured coded exosomes in the figure. The insert shows the interaction of the exosomes with the membrane of LEC which then internalise the exosomes containing ncRNA for interaction with cellular proteins and RNA/DNA. These abbreviations used are: LEC stands for lymphatic endothelial cell, CAF for cancer-associated fibroblast, MDSC for myeloid-derived suppressor cells and SMC for smooth muscle cell. This figure was created with BioRender.com.

Recently, miRNAs and lncRNAs have been found in exosomes in cancer (Figure 2). Exosomes are extracellular vesicles with diameters ranging from 30 to 150 nm which, in a tumour setting, can be secreted by tumour cells or immune or mesenchymal cells in the tumour microenvironment [58]. Specific ncRNAs are packaged in exosomes based on the presence of particular sequence motifs or structural configurations in these RNA molecules or interactions with certain RNA-binding proteins [59]. Uptake of exosomes by different cell types can be both selective (receptor-ligand interaction) and non-specific [60]; endothelial cells have been shown to uptake exosomes via receptor-mediated endocytosis [61]. miRNAs and lncRNAs in cancer-derived exosomes facilitate cell-cell communication which, in turn, can stimulate tumour growth, invasion, angiogenesis, lymphangiogenesis, metastasis and the reprogramming of the tumour microenvironment to favour tumour growth [62,63]. 

### 3.1. Role of Exosomal MiRNAs

Exosomal miRNAs are capable of regulating lymphangiogenesis and angiogenesis within the primary tumour and at metastatic sites (Table 1). Within a tumour, most exosomal miRNAs are considered to be produced by tumour cells and, when internalised by endothelial cells, some of these miRNAs can stimulate angiogenesis and/or lymphangiogenesis by repressing the expression of proteins that inhibit the main pathways that drive these processes [64,65,66]. Exosomal miRNAs have been shown to down-regulate several anti-angiogenic transcription factors in endothelial cells, and thereby initiate the angiogenic switch [24,67,68], or to repress inhibitors of the expression of VEGFA, a key inducer of angiogenesis. For instance, in gastric carcinoma, exosomal miR-155 and miR-130a, secreted by gastric cancer cells, have been shown in two studies to repress expression of the transcription factor c-MYB and thereby indirectly promote the expression of VEGFA [69,70]. Another example of exosomal miRNAs controlling key transcription factors to promote angiogenesis was reported by Mao and colleagues, who showed that exosomal miR-141 secreted by small-cell lung cancer cells downregulates the expression of the transcription factor KLF12 in endothelial cells thereby promoting angiogenesis in vivo [24]. Moreover, Ba and colleagues demonstrated that gastric cancer cells secrete exosomes containing miR-155 which stimulated the remodelling of blood vessels in vivo by targeting the anti-angiogenic transcription factor Forkhead box O3 (FOXO3a) [68].

Several research groups have also shown that tumour cells can secrete exosomal miRNAs which influence non-endothelial cell types in the tumour microenvironment such as fibroblasts and macrophages so they can, in turn, promote tumour angiogenesis and/or lymphangiogenesis. For instance, Fan and colleagues have shown that exosomal miR-210 derived from lung cancer cells induces the reprogramming of normal fibroblasts into cancer-associated fibroblasts (CAFs) by activating the JAK2/STAT3 pathway in these cells. Reprogrammed fibroblasts then upregulate the expression of several angiogenic factors, including VEGFA, which stimulate the remodelling of tumoural blood vessels [76]. Another example of tumour cells reprogramming non-endothelial cells in the tumour microenvironment was provided by two research groups in 2018 demonstrating changes in macrophages induced by cancer-derived exosomes. Specifically, Wang and colleagues showed that hypoxic pancreatic cancer cells secrete exosomes containing miR-301a which induced M2 polarization of macrophages by activating the PTEN/PI3Kgamma signalling pathway in these cells [74]. Furthermore, Yang and colleagues demonstrated that head and neck cancer cells promote M2 polarisation of macrophages by delivering miR-21-enriched exosomes to these cells which target mRNAs for a subunit of interleukin-12 (IL12A] and PDCD4, a protein involved in the initiation of protein translation [77]. M2 macrophages are considered activated tumour-associated macrophages and are known to produce inflammatory signals and express high levels of growth factors that stimulate angiogenesis and lymphangiogenesis, such as VEGFA and VEGFC [78].

In specific tumour settings, cells of the tumour microenvironment, including endothelial cells, can secrete exosomes that target tumour cells or other cell types and thereby indirectly regulate tumour angiogenesis. For instance, mesenchymal stem cells secrete exosomes containing miR-100 which inhibits angiogenesis by modulating the mTOR/HIF-1α/VEGFA signalling axis in breast cancer cells, thus reducing production of VEGFA by these cells [79]. Exosomal miR-126 has been shown to be secreted by chemotherapy-stimulated myeloid-derived suppressor cells, a heterogeneous population of cells which proliferates in response to the treatment of cancer with chemotherapy [80], and that has a strong ability to suppress T-cell responses [81]. miR-126-enriched exosomes target T2 helper immune cells and endothelial cells to promote both an inflammatory milieu and tumour angiogenesis in breast cancer mouse xenograft models [82]. 

### 3.2. Role of Exosomal LncRNAs

Recently, lncRNAs have been shown to be involved in cell-to-cell communication in the context of tumour angiogenesis [83]. lncRNAs can be packaged and secreted in exosomes, predominantly by tumour cells, and target the endothelium of blood vessels leading to the induction of angiogenesis [84,85]. Exosomal lncRNAs which have been internalised into the endothelial cells of blood vessels seem to regulate angiogenesis by behaving as competing endogenous RNAs, i.e., by binding endogenous miRNAs and preventing their function in BECs thereby derepressing expression of their targets involved in angiogenic signalling (Table 2). An example is UCA1 lncRNA which is secreted in exosomes by pancreatic cancer cells in hypoxic conditions and internalised by BECs. This lncRNA has been shown to sponge miR-206 in the blood vessel endothelium derepressing expression of its target AMOTL2 thereby activating the ERK1/ERK2 axis which is crucial for signal transduction downstream of VEGFA [16]. AMOTL2 belongs to the angiomotin family of membrane-associated scaffold proteins, and has been shown to play a significant role in the proliferation, tube formation and migration of endothelial cells via positively regulating the MAPK/ERK1/2 signalling pathway [86]. The derepression of AMOTL2 expression led to remodelling of BECs, angiogenesis and tumour growth in a mouse xenograft model of pancreatic cancer [25]. 

FAM225A lncRNA has been shown to promote oesophageal squamous cell carcinoma (OSCC) progression and angiogenesis by sponging miR-206 in BECs. Specifically, OSCC cells secrete high levels of exosomal FAM225A which can then be endocytosed by BECs wherein this lncRNA inhibits miR-206 leading to upregulation of the expression of miR-206 targets [87]. These include the angiogenic transcription factor FOXP1 and the transmembrane protein NETO2 which has been previously shown to activate the PI3K/Akt/NF-κB/Snail axis, and thus drive tumour progression, in the setting of gastric cancer [104]. As a result of the sponging of miR-206, FAM225A promotes angiogenesis and OSCC progression in vivo [87].

Another example of how exosomal lncRNAs regulate tumour angiogenesis is that chondrosarcoma cell-derived exosomes carry lncRNA RAMP2-AS1 which is delivered to BECs. Here, RAMP2-AS1 acts as a competing endogenous RNA by sponging miR-2355-5p thereby upregulating the expression of one of its targets, the VEGFA receptor VEGFR2, leading to activation of angiogenic signalling [88].

Some lncRNAs have been shown to directly upregulate the expression of VEGFA in endothelial cells thereby promoting angiogenesis. For example, it has been shown that exosomal lncRNA Hotair, produced by glioma cells, upregulates the expression of VEGFA in brain microvascular endothelial cells and thus stimulates their remodelling [84].

## 4. Relevance of Tumour-Derived Exosomal Non-Coding RNAs for Cancer Biomarkers and Therapeutic Interventions

### 4.1. Non-Coding RNAs as Cancer Biomarkers

Tumour-derived exosomes are found in a variety of body fluids including blood, tears, urine, milk, saliva and ascites [105], and the encapsulation of ncRNAs in these exosomes can help maintain their integrity in body fluids by protecting them from RNAses. Hence the analysis of tumour-derived exosomal ncRNAs is readily applicable for liquid biopsy. Liquid biopsy is the analysis of tumour biomarkers present in non-solid biological samples, primarily blood. Liquid biopsy, as opposed to traditional solid biopsy, has been increasingly used as a non-invasive diagnostic and molecular phenotyping tool to detect and diagnose tumours and provide information prior to treatment [106]. Liquid biopsy can also be used to monitor tumour evolution [107]. The ideal panel of tumour biomarkers for liquid biopsy must be able to identify the tumour type, its stage and, ideally, the best therapeutic treatment. Thanks to their specificity of sequence and function, and ability to withstand the relatively harsh conditions in the tumour microenvironment and circulation, cancer-derived exosomal ncRNAs in blood have the potential to meet these expectations [108]. An example of the use of ncRNAs in cancer diagnostics is provided by the company MiRXES (Singapore) which has developed a qPCR-based diagnostic test currently used for diagnosis of gastric cancer; the test analyses the levels of 12 microRNA biomarkers linked to gastric cancer and calculates a cancer risk score for each patient using an algorithm that has been clinically validated (https://mirxes.com/gastroclear/ (accessed on 9 February 2021)) [109].

We envisage a scenario in which cancer-derived exosomal ncRNAs that promote tumour angiogenesis and lymphangiogenesis (such as those discussed in previous sections of this article) are included in panels of ncRNA biomarkers for liquid biopsy to detect and stage cancers in the clinic. The purpose of monitoring ncRNAs that promote angiogenesis and lymphangiogenesis would be to provide information about the propensity of detected tumours to metastasize, via blood vessels or lymphatics, to distant sites in the body which is the most lethal aspect of cancer for most patients. We acknowledge there is considerable work that still needs to be done to clinically validate specific exosomal ncRNAs as biomarkers of cancer metastasis.

### 4.2. Non-Coding RNAs and Therapeutics

In this article we have focussed on ncRNAs which promote angiogenesis, lymphangiogenesis and metastasis in cancer. In contrast, there are a range of ncRNAs which are known to be anti-angiogenic or anti-lymphangiogenic and could therefore form the basis of therapeutic strategies to restrict tumour growth and spread [15,110,111]. Such ncRNAs could be packaged in liposomes and delivered to patients, via injection, to restrict tumour angiogenesis and lymphangiogenesis, and thereby inhibit metastatic spread. This strategy is speculative at this time, however, the potential feasibility of delivering synthetic ncRNAs in vivo is illustrated by the approach of using lipid-nanoparticle formulated, nucleoside-modified synthetic RNA in the clinic for immunization to combat coronaviruses such as SARS-CoV-2, as exemplified by the BNT162b2 mRNA Covid-19 vaccine [112].

There are a range of non-cancer clinical settings in which it could be beneficial to promote angiogenesis and/or lymphangiogenesis such as wound healing, cardiovascular diseases and lymphoedema. ncRNAs which promote angiogenesis and lymphangiogenesis, such as those discussed in this article, could be exploited for these purposes by employing lipid-nanoparticle formulated, nucleoside-modified synthetic RNA. The potential of such a strategy has been exemplified by recent animal studies in which lymphangiogenesis was successfully stimulated in a therapeutic setting by delivering exosomal miRNAs, derived from adipose-derived stem cells (ADSCs), to LECs. ADSCs are multipotent cells located in adipose tissue that have been used in therapeutic settings [113] because of their stability in different tissue environments and their capacity to secrete growth factors and exosomes [114]. Wang and colleagues have shown that ADSCs treated with VEGFC produce high levels of exosomal miR-132 which in turn promoted the remodelling of LECs [71]. Furthermore, An and colleagues showed that ADSCs naturally secrete high levels of exosomal miR-21 which stimulated BEC remodelling and therefore could potentially be used to stimulate therapeutic angiogenesis [96]. 

## 5. Concluding Remarks

While non-coding RNAs do not encode proteins they do play a critical role in regulating the levels of many cellular and extracellular proteins [115], and thus influence key signalling pathways involved in complex biological processes such as angiogenesis, lymphangiogenesis and metastasis in cancer which have been previously shown to be regulated by several growth factors and their cognate receptors. The broad role of non-coding-RNAs in biology is now well established and their impact on human disease is undergoing intensive characterisation. The presence of non-coding RNAs in cancer-derived exosomes allows them to actively participate in cell-to-cell communication in the tumour microenvironment and, potentially, at more distant sites in the body. It is already clear that cancer-derived exosomal non-coding RNAs can promote tumour angiogenesis and lymphangiogenesis by altering gene expression in a range of cell types including endothelial cells (i.e., BECs and LECs), fibroblasts and macrophages. Hence the regulatory role that non-coding RNAs play in tumour angiogenesis and lymphangiogenesis can be considered multi-dimensional.

Our current insight into the regulation of tumour angiogenesis and lymphangiogenesis by non-coding RNAs can be exploited for development of new cancer biomarkers and therapeutics, as discussed here. However, further research is required for this to occur. In particular, specific non-coding RNAs which promote tumour angiogenesis and/or lymphangiogenesis will need to be clinically validated as useful biomarkers of metastasis before being incorporated in liquid biopsy strategies for detecting and staging cancers. The use of anti-angiogenic or anti-lymphangiogenic non-coding RNAs as therapeutics to restrict tumour growth and spread seems attractive, particularly in light of recent regulatory approvals for vaccines based on lipid-nanoparticle formulated, nucleoside-modified RNA. A key challenge for this approach will be to determine the best anti-angiogenic/anti-lymphangiogenic non-coding RNAs to use for restricting cancer metastasis, which will need to be carefully assessed in clinically relevant animal models of cancer.

## Figures and Tables

**Table 1:** Regulation of Lymphatic Remodelling by exosome-derived miRNAs.

Cells Producing Exosomes	miRNA in Exosomes	Target Gene	Role of MiRNA in Promoting Lymphangiogenesis	Recipient Cells	Reference
Adipose-derived stem cells	miR-132	Smad-7	Regulating TGF-β pathway	LECs	[71]
Cervical squamous cell carcinoma cells	miR-221-3p	Vasohibin-1	Regulating an inhibitor of lymphangiogenesis implicated in microtubule dynamics	LECs	[72]
Hepatocellular carcinoma cells	miR-296	EAG1	Inhibiting VEGFA expression	LECs	[73]
Hypoxic pancreatic cancer cells	miR-301a-3p	PTEN	Promoting M2 polarization of macrophages by activation of the PTEN/PI3Kγ pathway	Macrophages	[74]
Cervical squamous cell carcinoma cells	miR-142-5p	ARID2	Inducing IDO expression via ARID2–DNMT1–IFN-γ signalling to suppress CD8+ T cells	LECs	[72,75]

ARID2 = AT-rich interactive domain-containing protein 2; DNMT1 = DNA (cytosine-5)-methyltransferase 1; EAG1 = Ether-à-go-go-1; IFN-γ = Interferon gamma; LECs = Lymphatic endothelial cells; PI3Kγ = Phosphatidylinositol 3-kinase-gamma; PTEN = Phosphatase and tensin homolog; TGF-β = Transforming growth factor beta; VEGFA = Vascular endothelial growth factor A.

**Table 2:** Regulation of angiogenic remodelling by exosome-derived non-coding RNAs.

Cells Producing Exosomes	ncRNA in Exosomes	mRNA/miRNA Target	Role of ncRNA in Promoting Angiogenesis	Recipient Cells	Reference
Hypoxic pancreatic cancer cells	UCA1 lncRNA	miR-96-5p	Sponging miR-96-5p thus derepressing its target AMOTL2 thereby activating ERK1/ERK2 axis	HUVECs	[25]
Oesophageal squamous cell carcinoma cells	FAM225A lncRNA	miR-206	Sponging miR-206 thus derepressing its targets NETO2 and FOXP1 thereby activating PI3K/Akt/NF-κB/Snail axis	HUVECs	[87]
Chondrosarcoma cells	RAMP2-AS1 lncRNA	miR-2355-5p	Sponging miR-2355-5p thus derepressing its target VEGFR2 thereby increasing angiogenic cell surface receptors	HUVECs	[88]
Glioma cells	POU3F3 lncRNA	bFGF, VEGFA, bFGFR	Increasing the expression of bFGF, VEGFA and bFGFR in endothelial cells	HBMECs	[85]
Glioma cells	HOTAIR lncRNA	VEGFA	Increasing the expression of VEGFA in endothelial cells	HBMECs	[84]
Glioma cells	CCAT2 lncRNA	VEGFA, TGF-β, Bcl-2, and Bax	Increasing the expression of VEGFA and other angiogenic signalling in endothelial cells plus decreasing apoptosis	HUVECs	[89]
Small-cell lung cancer cells	miR-141	KLF12	Repressing an anti-angiogenic transcriptional factor	HUVECs	[24]
Glioma cells	miR-148a-3p	ERRFI1	Repressing an anti-angiogenic cell surface receptor	HUVECs	[90]
Oesophageal squamous cell carcinoma cells	miR-210-3p	EphrinA3	Repressing ephrinA3 and therefore activating PI3K/AKT signalling	HUVECs	[91]
Glioblastoma multiforme cells	miR-182-5p	KLF2 and KLF4	Repressing anti-angiogenic transcription factors	HUVECs	[67]
Lung cancer cells	miR-210	TET2	Reprogramming normal fibroblasts into CAFs	Fibroblasts	[76]
Epithelial ovarian cancer cells	miR-141-3p	SOCS5	Repressing an inhibitor of the JAK/STAT3 and NF-κB signalling pathways	HUVECs	[64]
Non-small cell lung cancer cells	miR-619-5p	RCAN1.4	Repressing an inhibitor of the calcineurin/NFAT pathway	HUVECs	[65]
Gastric carcinoma cells	miR-130a	c-MYB	Repressing an inhibitor of the expression of VEGFA	HUVECs	[70]
Gastric carcinoma cells	miR-155	c-MYB	Repressing an inhibitor of the expression of VEGFA	HUVECs	[69]
Gastric carcinoma cells	miR-155	FOXO3a	Repressing anti-angiogenic transcription factors	HUVECs	[68]
Nasopharyngeal carcinoma cells	miR-17-5p	BAMBI	Repressing an inhibitor of the VEGFA/AKT axis	HUVECs	[66]
HUVECs	miR-126	IRS1, VEGFA and EGFL7	Targeting crucial factors involved in this process	Malignant mesothelioma cells	[92]
Ovarian cancer cells	miR-205	PTEN	Repressing a phosphatase that negatively regulates AKT	HUVECs	[93]
Gastric cancer cells	miR-135b	FOXO1	Repressing anti-angiogenic transcription factors	HUVECs	[94]
Adipose-derived stem cells	miR-199-3p	Sema3A	Driving the migration of endothelial tip cells and their sprouting	HUVECs	[95]
Adipose-derived stem cells	miR-21	PTEN	Repressing a phosphatase that negatively regulates AKT	HUVECs	[96]
Osterosarcoma cells	miR-21	PTEN	Repressing a phosphatase that negatively regulates AKT	HUVECs	[97]
Glioma stem cells	miR-26a	PTEN	Repressing a phosphatase that negatively regulates AKT	HUVECs	[98]
Glioma stem cells	miR-21	Not identified	Upregulating VEGFA expression in endothelial cells	HUVECs	[99]
Cervical squamous cell carcinoma cells	miR-221-3p	THBS2	Repressing a potent endogenous inhibitor of angiogenesis	HUVECs	[100]
Glioma cells	miR-9	COL18A1, THBS2, PTCH1 and PHD3	Repressing endogenous inhibitors of angiogenesis and initiating HIF-1α/VEGF signal transduction	HUVECs	[101]
Nasopharyngeal carcinoma cells	miR-23a	TSGA10	Repressing TSGA10, a novel inhibitor of angiogenesis	HUVECs	[102]
Mesenchymal stem cells	miR-100	mTOR	Inducing expression of VEGFA in tumour cells	Breast cancer cells	[79]
Hypoxic lung cancer cells	miR-23a	PHD1, PHD2 and ZO-1	Initiating HIF-1α/VEGFA signal transduction, and promoting vascular permeability by destabilising cellular junctions	HUVECs	[103]
Hepatocellular carcinoma cells	miR-210	SMAD4 and STAT6	Repressing anti-angiogenic transcription factors and signal transducers	HUVECs	[62]
Head and neck cancer cells	miR-21	PTEN, PDCD4 and IGFBP3	Stimulating M2 polarization of tumour-associated macrophages	Macrophages	[77]
Myeloid derived suppressor cells	miR-126a	Not identified	Promoting an inflammatory milieu that leads to metastasis	CD4^+^ T-helper cell type-2	[82]

BAMBI = BMP and Activin receptor Membrane Bound Inhibitor; CAFs = cancer-associated fibroblasts; COL18A1 = Collagen alpha-1(XVIII) chain; EGFL7 = EGF-like domain 7; ERRFI1 = ERBB Receptor Feedback Inhibitor 1; FOXO = Forkhead Box; HBMECs = Human brain microvascular endothelial cells; HIF-1α = Hypoxia-inducible factor 1-alpha; HUVECs = Human umbilical vein endothelial cells; IGFBP3 = Insulin-like growth factor-binding protein 3; IRS1 = insulin receptor substrate 1;KLF = Kruppel Like Factor; mTOR = mammalian target of rapamycin; PDCD4 = Programmed Cell Death 4; PHD = Prolyl hydroxylase; PTCH1 = Patched 1; PTEN = Phosphatase and tensin homolog; RCAN = Regulators of the calcineurin; Sema3A = Semaphorin 3A; SOCS5 = Cytokine signaling 5; STAT4 = Signal transducer and activator of transcription 4; TET2 = Tet methylcytosine dioxygenase 2; THBS2 = Thrombospondin-2; TSGA10 = Testis Specific 10; VEGFA = vascular endothelial growth factor A; VEGFR2 = Vascular endothelial growth factor receptor 2; ZO-1 = Zonula occludens-1.
